# Correction to [Metal‐Catalyzed Abiotic Cleavage of C=C Bonds for Effective Fluorescence Imaging of Cu(II) and Fe(III) in Living Systems]

**DOI:** 10.1002/advs.202505490

**Published:** 2025-04-15

[C. Wang, D. Chen, Z. Wei, J. Tan, C. Wu, X. Zhang, Metal‐Catalyzed Abiotic Cleavage of C=C Bonds for Effective Fluorescence Imaging of Cu(II) and Fe(III) in Living Systems. Adv. Sci. 2025, 12, 2412407.]

https://doi.org/10.1002/advs.202412407

In Figure 5, the figure was incorrect:


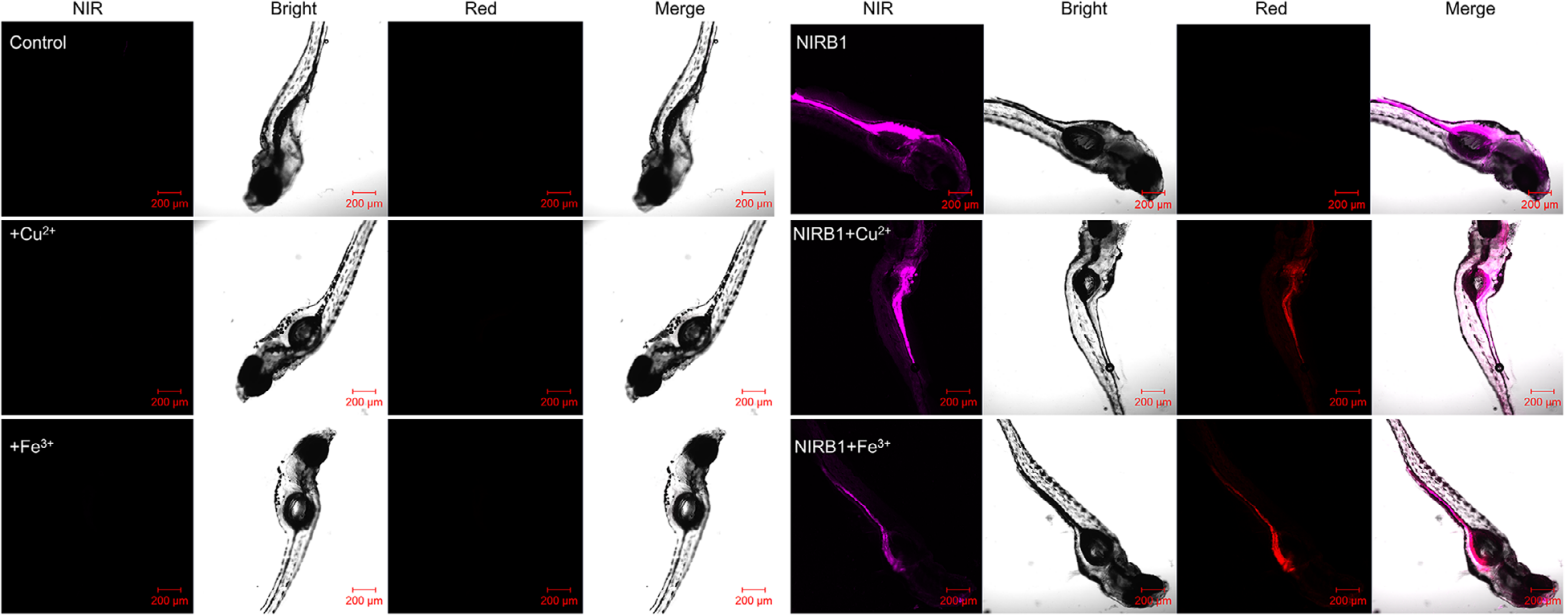


This should be corrected as follows:


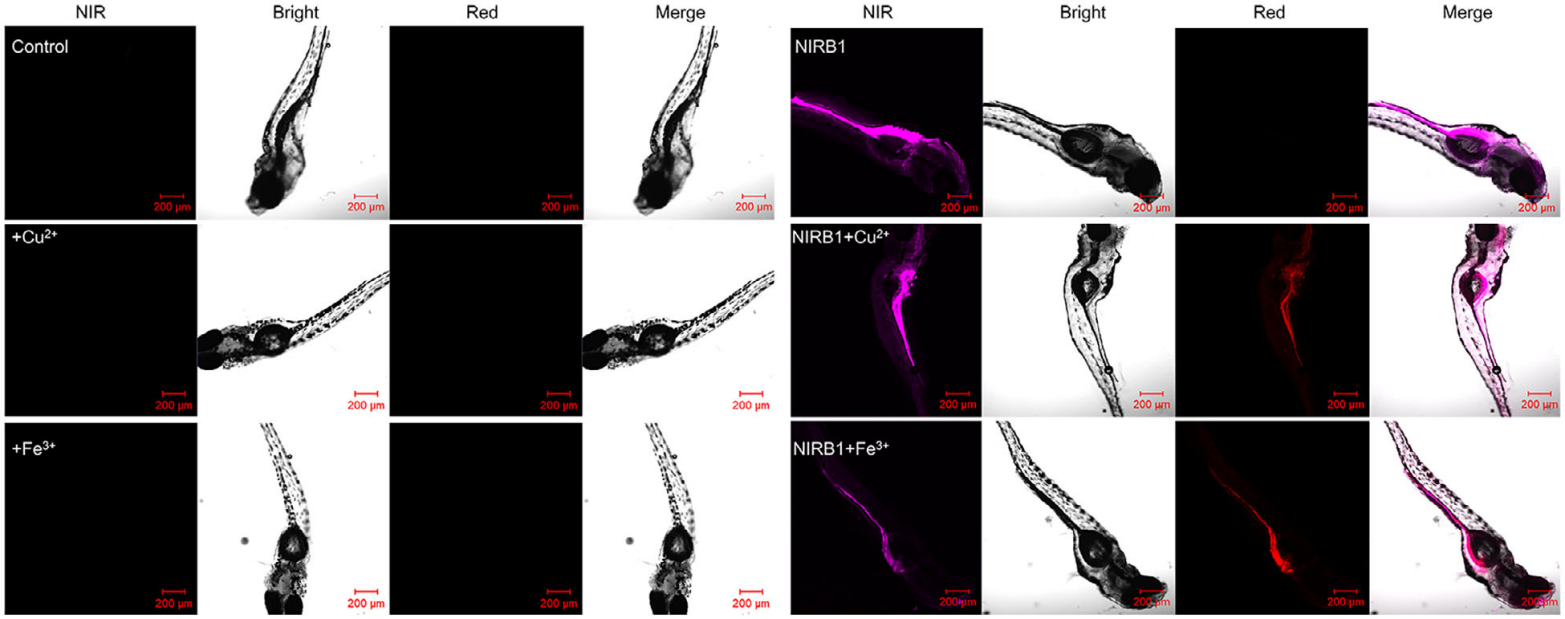


This correction does not affect any conclusions of the paper. We apologize for this error.

